# Food-related impulsivity assessed by longitudinal laboratory tasks is reduced in patients with binge eating disorder in a randomized controlled trial

**DOI:** 10.1038/s41598-021-87231-w

**Published:** 2021-04-15

**Authors:** Kathrin Schag, Elisabeth J. Leehr, Paolo Meneguzzo, Peter Martus, Stephan Zipfel, Katrin E. Giel

**Affiliations:** 1 Department of Psychosomatic Medicine and Psychotherapy, Medical University Hospital Tübingen, Osianderstraße 5, 72076 Tübingen, Germany; 2 Competence Center for Eating Disorders Tübingen (KOMET), Tübingen, Germany; 3 Department of Psychiatry and Psychotherapy, University of Münster, Münster, Germany; 4 Department of Neurosciences, University of Padova, Padova, Italy; 5 Institute for Clinical Epidemiology and Applied Biostatistics, Medical University Hospital Tübingen, Tübingen, Germany

**Keywords:** Cognitive neuroscience, Psychiatric disorders, Nutrition, Therapeutics

## Abstract

Food-related impulsivity, i.e. a food-related attentional bias proposed to be due to increased reward sensitivity and diminished inhibitory control, has been cross-sectionally associated with binge eating disorder. To analyze changes in food-related impulsivity, we implemented longitudinal analyses of objective laboratory tasks in a randomized controlled trial called IMPULS. Patients who attended an impulsivity-focused group intervention (IG N = 31) and control patients who did not take part in the intervention (CG N = 25) were compared before (T0) and after the intervention period (T1) and at three months follow-up (T2). Patients’ impulsive gaze behavior towards food vs. neutral stimuli was measured in two eye tracking paradigms, one addressing reward sensitivity and another addressing inhibitory control. Initial fixations of food vs. neutral stimuli were increased at T0 (IG: *p* = .014, CG: *p* = .001), but not at T1 and T2 in IG (T1: *p* = .178, T2: *p* = .203) and in CG after Bonferroni correction only at T2 (T1: *p* = .031, T2: *p* = .002). Patients from IG increased dwell time on neutral stimuli at T1 contrary to patients from CG (*p* = .016) and rated the presented food stimuli as less positive (e.g. pleasantness *p* < .001 at T1 and T2). A possible explanation for this observation is reduced reward sensitivity, which implies a short-term treatment effect. Both groups showed improvement in inhibiting eye movements towards food and neutral stimuli over time (i.e. first saccade errors overall *p* < .001, second saccade errors overall *p* < .003). This could indicate increased inhibitory control due to training effects from the study paradigm. The results suggest that food-related impulsivity represents an underlying mechanism of BED and that it is modifiable by cognitive behavioral interventions.

## Introduction

Binge eating disorder (BED) is characterized by recurrent binge eating episodes, accompanied by experienced loss of control^1^. BED represents a public health concern and the prevalence rate of obesity in patients with BED is highly increased^2,3^. Like other eating disorders, the etiology and maintenance of BED is regarded as multifactorial, with a special focus on several neurocognitive deficits in executive functioning, such as inhibitory control and attentional bias^4–6^. Attentional bias is provoked by highly emotional environmental stimuli, e.g. threatening or rewarding ones, and can result from learning mechanisms^7^. An attentional bias can be concisely defined as a difficulty in disengaging from and/or over focusing on salient stimuli^4,7^. Inhibitory control is a vital part of self-regulation processes. It includes the ability to suppress a behavioral response (proactive inhibition) or to stop an already initiated behavioral response (reactive inhibition)^4^. Systematic reviews of cross-sectional studies including eye tracking research report an increased attentional bias towards food-related stimuli as well as decreased inhibitory control in patients with BED, particularly in food-related paradigms^4,5,8^. These observed deficits in both neurocognitive domains might be best understood on the basis of an underlying personality trait, i.e. impulsivity^9,10^.

Impulsivity consists of two main factors: increased reward sensitivity (reflected by an increased attentional bias towards rewarding stimuli), and decreased inhibitory control^11,12^. Thus, binge eating can be understood as impulsivity-driven eating behavior with a) increased reward sensitivity concerning food stimuli represented by increased cue reactivity and craving, and b) decreased inhibitory control represented by a perceived loss of control while eating^13^. Similarly, Stojek et al. proposed that “binge eating develops as a result of oversensitivity to rewarding properties of food and is captured by AB (attentional bias) to food”^5,p^^. 13^. Additionally, Kessler et al.^14^ proposed a neurobiological model for BED with maladaptation in the corticostriatal circuitry, which incorporate reward, motivation and inhibitory control as underlying constructs.

Despite growing cross-sectional evidence in this field (see above), longitudinal research with objective measures is lacking. In order to identify risk and maintenance factors in BED and elucidate the trajectories of impulsive behaviors, e.g. if such neurocognitive impairments change hand in hand with symptomatology, longitudinal evidence is vital. To date, only one study has examined the predictive value of laboratory impulsivity tasks on binge eating frequency after BED treatment. Results showed that, while laboratory tasks did not predict treatment outcome^15^, several self-report measures of trait impulsivity did predict binge eating frequency^15–18^. Sharma and colleagues^19^ concluded from their meta-analysis that such conflicting results could be due to low correlations between self-reported trait impulsivity and laboratory impulsivity tasks. Further, they highlighted the importance of exploring the trajectories of laboratory assessed impulsivity. Laboratory tasks were proposed to measure rather the current state of impulsive behaviors, while self-report measures reflected rather enduring impulsivity traits^20^.

In particular, there is hardly any evidence concerning the potential impact of eating disorder interventions on laboratory assessed impulsivity. One laboratory study with eating disorder patients indicated that decreased eating disorder pathology is accompanied by a decreased attentional bias after patients underwent cognitive behavioral treatment (CBT)^21^. In a pilot eye tracking study carried out by our group^22^, patients with BED showed reduced inhibitory control deficits and binge eating frequency after an inhibitory control training. However, the same observations were also found in the control group after a free viewing paradigm. In a first randomized controlled trial in patients with obesity where one third of the patients suffered additionally from BED^23^, a group intervention focusing on inhibitory control and emotion regulation in combination with an computer-assisted inhibitory control training led to an increased reduction of inhibitory control deficits, as compared to a control group who underwent CBT. Taken together, though these preliminary studies used different laboratory tasks, treatments or training programs and diverse patient groups, it seems plausible that an intervention with a special focus on impulsivity is likely to be efficacious in reducing food-related impulsivity in patients with BED. We hypothesize such an intervention would be able to improve not only the behavioral symptoms, but also underlying mechanisms, i.e. patients’ attentional biases as well as inhibitory control capacities measured by laboratory tasks.

Recently, we completed the randomized controlled IMPULS trial, which investigated the efficacy of a CBT-oriented impulsivity-focused group intervention for BED. We compared patients from the treatment group who received the IMPULS treatment (IG) with a control group where patients received no treatment (CG) besides self-monitoring of binge eating and other impulsive behaviors. In this trial^24^, we showed that binge eating frequency and global eating disorder pathology are decreased in IG after treatment. However, analysis on the primary outcome, i.e. the frequency of binge eating episodes in the past four weeks, failed, as short-term improvement was also observed in CG. In the current planned sub-study^13^, we investigate whether both components of food-related impulsivity, assessed longitudinally by two eye tracking tasks, will be reduced after the IMPULS treatment (primary outcome). More precisely, we hypothesize primarily that food-related attentional bias and inhibitory control deficits in patients with BED before treatment (T0) will be reduced after treatment (T1) in IG contrary to CG. We additionally expect this effect to be more pronounced in food related stimuli as compared to neutral stimuli. Secondary hypotheses include further group, time and other interactional effects concerning the eye tracking assessments at T1, as well as at three months follow-up (T2). Further, we hypothesize that patients from IG will significantly reduce self-reported valence assigned to food stimuli at T1 and T2 as compared to CG, which indicates decreasing reward sensitivity. We additionally explore associations between symptom levels and potential underlying mechanisms.

## Methods

This study was performed in a subgroup of patients enrolled in the randomized controlled IMPULS trial^24^, German Clinical Trials Register, ID: DRKS00007689, date of first registration 14/01/2015. It has been approved by the ethics committee of the Medical Faculty of the Eberhard Karls University Tübingen and the University Hospital Tübingen, Germany. All procedures were in accordance with the ethical standards of the declaration of Helsinki. Informed consent was obtained from all participants included in the study. The Institute for Clinical Epidemiology and Applied Biometry, Tübingen, Germany conducted the stratified randomization and provided support in data analysis. The full study protocol of the IMPULS trial^13^, main clinical outcomes,^24^ as well as the IMPULS intervention^25^ have been previously published.

### Participants

**Figure 1:**
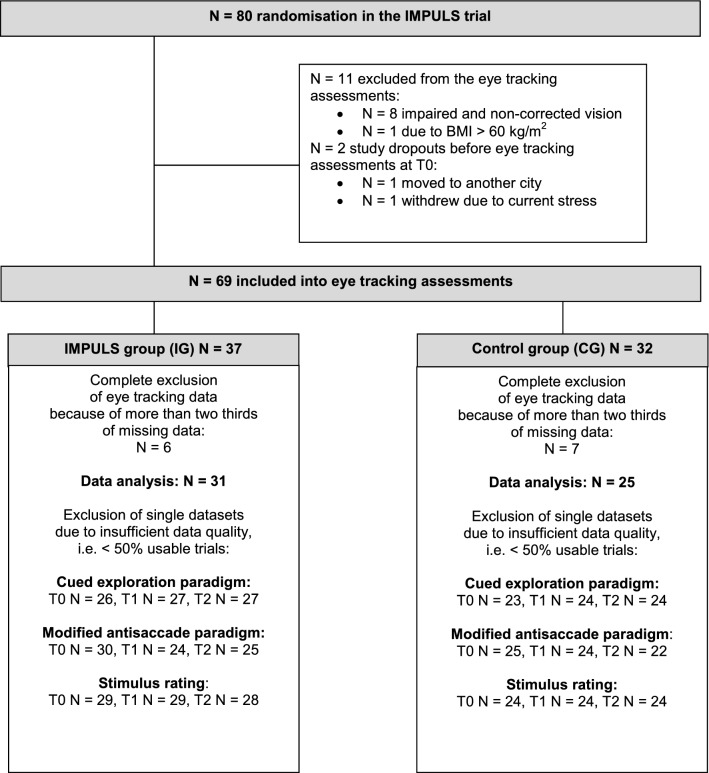
Patient flow chart of the eye tracking analysis in the IMPULS trial.

From 80 randomized patients in the IMPULS trial^24^ with BED, a subgroup of 69 patients participated in the eye tracking assessments presented here. The patient flow is reported in Fig. 1.

Inclusion criteria for the main IMPULS trial included a BED diagnosis according to DSM-5^1^, being at least 18 years of age, and a written informed consent. Exclusion criteria included severe psychiatric disorders (e.g. psychosis, addictions), pregnancy, and somatic conditions with influence on eating behavior (e.g. modified medication for diabetes or thyroid diseases). Additionally, patients were excluded from eye tracking assessments if they had impaired or non-corrected vision (N = 8), were physically unable to participate (N = 1, due to having BMI > 60) or dropped out from the main trial before eye tracking assessments were conducted (N = 2). Finally, 37 patients from IG and 32 patients from CG were investigated, i.e. 86% from the sample of the main IMPULS trial.

### Experimental eye tracking paradigms and stimulus material

**Figure 2:**
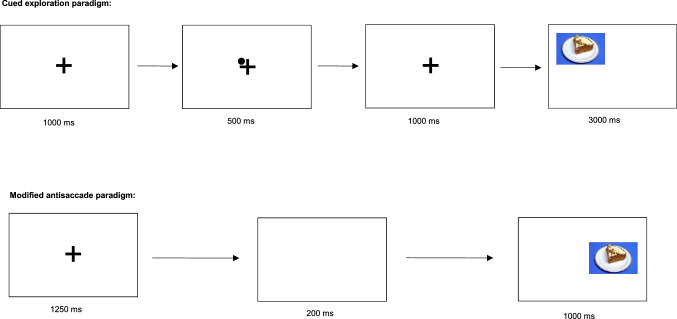
Schematic presentation of the cued exploration paradigm and the antisaccade paradigm. © Schag et al. (2015)^13^, slightly modified.

We used two eye tracking paradigms with high-caloric food vs. neutral stimuli to assess impulsive gaze behavior. The paradigms, as well as an example of the stimuli (i.e. cake), are presented at Fig. 2. The food stimuli presented here consisted of 40 high-caloric savory or sweet foods such as potato chips, jelly babies, pizza and pancakes. The neutral stimuli were household items such as rubber bands, pens, brushes or sponges. The two categories of stimuli were matched in terms of form, brightness, color and contrast. Similar eye tracking paradigms with the same stimuli have been implemented previously in eating disorder studies^22,26,27^.

We assessed reward sensitivity concerning food stimuli with the cued exploration paradigm. In this paradigm, patients were instructed to visually explore 40 stimulus pairs, consisting of food vs. neutral stimuli. Each pair was presented for 3000 ms. Before each trial, a fixation cross and a dot in one corner of the cross were presented. The position of the dot is an endogenous cue^28^ which indicated where the next food stimulus will be displayed. Two dependent variables were assessed: the percentage of initial fixation position on the food vs. neutral stimuli, and the percentage of dwell time on the food vs. neutral stimuli. Both variables imply a top-down controlled conscious attentional bias, with relation to attention allocation and disengagement respectively^5^.

In the modified antisaccade paradigm, we assessed food-related inhibitory control. Here, patients were instructed to look away from the high-caloric food or the neutral stimuli. Both categories of stimuli were presented in randomized order as single pictures for 1000 ms at either the left or right side of the screen. Patients completed 160 trials. As dependent variables, we examined the percentage of saccade errors for food vs. neutral stimuli, i.e. when the participants failed to look away from the stimuli. Errors of first saccades correspond to proactive behavioral inhibitory control, while errors of second saccades represent reactive response inhibition and corrective behavior^4,29^.

We executed the paradigms via Java and recorded the data with a IViewX Hi-Speed system from Sensomotoric Instruments that has a 500 Hz sampling rate and 0.25–0.5° gaze position accuracy and used IViewX 2.8 software^30^. We analyzed the data with BeGaze 3.7 software^31^. Unrecorded trials and trials in which instructions were not adhered to (e.g. not fixating the cross between trials, premature response < 80 ms, delayed response > 800 ms) were not considered. Data was aggregated over all trials for each patient.

### Procedure

At baseline (T0), we assessed sociodemographic data, BED diagnosis with the standardized Eating Disorder Examination interview (EDE)^32^, and mental comorbidities with the Structured Clinical Interview for DSM-IV Disorders (SCID I)^33^. BMI (kg/m^2^) was calculated from measured height and body weight. Eating pathology was assessed with the Eating Disorder Examination Questionnaire (EDE-Q)^34^, and depression with the Beck Depression Inventory (BDI II)^35^. Subsequently, patients were randomized into the two groups.

At a second baseline appointment, impulsive gaze behavior was assessed with the two eye tracking paradigms described earlier. To prevent homoeostatic effects, patients were instructed to fast overnight and received a standardized breakfast directly before taking part in the experiment. Additionally, participants rated the presented food stimuli on a Likert scale ranging from − 5 to + 5 concerning pleasantness, palatability, wanting (currently) and liking (in general); neutral stimuli were rated concerning pleasantness according to Leehr and colleagues^36^. We also assessed patients’ current food craving with the Food Craving Questionnaire State (FCQ-S)^37^. Tait impulsivity was assessed with the Barrat Impulsiveness Scale (BIS-15)^38^ as well as the Behavioral Inhibition System/Behavioral Activation System (BIS/BAS)^39^.

After the completion of baseline measures, patients in IG took part in the IMPULS treatment (see below) while patients in CG received no treatment in the time span between T0 and T1. After the intervention period, the diagnostic and eye tracking appointments were repeated at the end of treatment (T1) and at three-months follow-up (T2).

### Treatment

The IMPULS treatment is comprised of an outpatient cognitive behavioral group treatment for 4–6 patients, which consists of eight weekly sessions (90 min each). According to the standardized IMPULS manual, the treatment utilizes interventions with special focus on the reduction of impulsive eating behavior, i.e. binge eating. The treatment comprises two parts: first, the identification of risky situations which could trigger a binge eating episode, as well as the development of self-control strategies aimed at preventing binge eating; second, patients engaged in food cue exposure with response prevention, i.e. patients are exposed to their individually chosen binge food and learn to withhold from eating it. More information on the IMPULS treatment is reported in^13,24,25^.

Additionally, IG and CG engaged in weekly self-observations, where they reported the frequency of binge eating episodes, as well as other impulsive behaviors according to ICD-10^40^ and DSM-5^1^. Patients also reported occurrences where they successfully inhibited such behaviors, as well as alternative behaviors they executed.

### Statistical analysis

The sample size for the main IMPULS trial was determined to 35 patients per group with a power of 80% at a two-sided α level of 0.05 according to the primary outcome of this trial, i.e. the binge eating frequency after four weeks assessed by the EDE^13^. Due to expected drop outs, we randomized 80 patients into the trial (see Fig. 1). Of these, a subgroup of 69 patients participated in the secondary sub study presented here. With this sample size, an ex post calculation results in detectable effect sizes of 0.69 with power 80% and level of significance 0.05 (two-sided). However, due to Bonferroni correction with factor 2 (see below) the detectable effect size increased to 0.76 (nQuery software, release 7).

From the N = 37 participants in IG and N = 32 in CG, patients who had more than two-thirds of missing data due to study drop out (N = 10) or insufficient data quality (N = 3) were discarded from the analyses. As a result, data from N = 31 participants in IG and N = 25 in CG were analyzed (see Fig. 1). Further, single datasets with low data quality, i.e. less than 50% of usable trials, were excluded from data analysis. As such, 73% of the potentially available data was analyzed in the cued exploration paradigm, and 73% in the modified antisaccade paradigm (see Fig. 1). Concerning the stimulus rating, single datasets were missing, so 76% of the potentially available data was analyzed (see Fig. 1). Additionally, due to recording problems in the stimulus rating, there was 40–60% data loss in N = 7 patients at T0. We decided to keep these data in the analyses, as means and standard deviations were consistent with the complete data sets at T0 (food stimuli: pleasantness *p* = 0.79, palatability *p* = 0.77, wanting *p* = 0.46, liking *p* = 0.48; neutral stimuli: pleasantness *p* = 0.65).

All data was analyzed with the Statistical Package of Social Sciences version 25 (SPSS). The study arms were compared with T-tests or Mann–Whitney-U tests, if data was not normally distributed, and Fishers Exact tests were used for binary data. The eye tracking data and stimuli ratings were analyzed with Generalized Estimating Equations (GEE)^41^. We changed analysis strategy from analysis of variance^13^ to GEE to be consistent with our main paper^24^. For each variable, we used a linear model with normal distribution and identity link, because they were normally distributed (skewness and kurtosis between -1 and + 1). The variable “second saccade errors (%)”was normally distributed after a log transformation, and the variable “neutral stimuli rating” was normally distributed after a symmetrical log transformation^42^. In the GEEs of the eye tracking data, the factors group, measurement point and stimulus were included. Subsequently, the respective main effects and interaction effects were analyzed. In the GEEs of the stimulus rating variables, the factors group and measurement point were included. Statistical significance was set at a two-sided *α*-level of 0.05. This was corrected for multiple comparisons concerning the primary and secondary hypotheses with the factor 2 on a two-sided *α*-level of 0.025, as two different factors of impulsivity were analyzed with equal emphasis. For all GEEs, we report Wald *Χ*^*2*^ as test statistic, regression coefficients (B) as effect sizes, and 95% Wald confidence intervals (CI) in order to remain consistent with our main paper^25^.

Additionally, Pearson correlations were computed to analyze associations (pooled for study arm and measurement point) between the four eye tracking variables concerning food stimuli with eating disorder pathology (EDE-Q total score, FCQ-S total score), BMI, the four stimulus ratings concerning food stimuli and trait impulsivity (BIS-15 total score, BIS total score and BAS total score). This resulted in 40 correlations, so that statistical significance was Bonferroni-corrected at an *α*-level of 0.00125.

## Results

### Sample characteristics

The sample characteristics are presented at Table 1. The sample consisted mainly of females (M = 86%) in the middle ages (M = 39 years) with severe obesity (BMI M = 36.7). There were no group differences at baseline. However, the FCQ-S total score differed nearly significant between groups at T0 (*p* = 0.08), though it did not differ between groups at T1 (*p* = 0.56) and T2 (*p* = 0.90). This has been considered in a sensitivity analysis (see below).

**Table 1:** Sample characteristics of the IMPULS group (IG) and the control group (CG) at baseline (T0).

	IG (N = 31)	CG (N = 25)	*p*
Female sex, *n (%)*	27 (87%)	21 (84%)	1.0
Age, *M (SD)*	38.2 (12.1)	39.8 (13.8)	.60
Nationality, *n (%)*			
German	24 (77%)	22 (88%)	.49
Other	7 (23%)	3 (12%)	
Education (school graduation), *n (%)*			
No/low	7 (23%)	2 (8%)	.17
Medium/high	24 (77%)	23 (92%)	
BMI (kg/m^2^), *M (SD)*	35.9 (9.4)	37.5 (9.9)	.55
Current mental comorbidities acc. to SCID I, *n (%)*	11 (36%)	8 (32%)	1.0
BDI II total score, *M (SD)*	14.7 (12.4)	12.6 (8.0)	.46
EDE-Q total score, *M (SD)*	2.8 (1.0)	2.8 (1.1)	.92
FCQ-S total score, *M (SD)*	39.2 (14.0)	32.2 (14.8)	.08
BIS-15 total score, *M (SD)*	33.9 (7.7)	35.0 (7.7)	.59
BIS total score, *M (SD)*	3.2 (.6)	3.1 (.4)	.89
BAS total score, *M (SD)*	3.0 (.4)	3.1 (.3)	.81

*BAS* Behavioral Activation System, *BIS* Behavioral Inhibition System, *BIS-15* Barratt Impulsiveness Scale (short version), *BDI II* Beck Depression Inventory (second version), *EDE-Q* Eating Disorder Examination Questionnaire, *FCQ-S* Food Craving Questionnaire State, *SCID I* Structured Clinical Interview for DSM-IV Disorders, Axis I.

### Cued exploration paradigm

**Figure 3:**
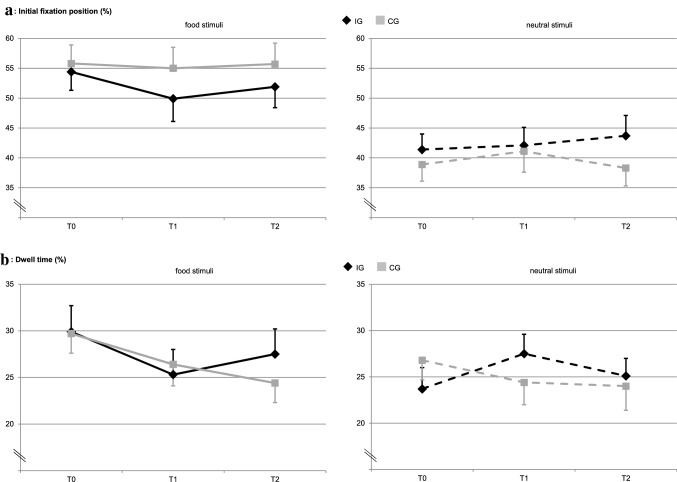
Eye tracking data of the cued exploration paradigm. Panel **(a)**: M (SE) of the initial fixation position (%), and Panel **(b)**: M (SE) of the dwell time (%) for food and neutral stimuli by treatment group (IG, CG) and measurement point (T0, T1, T2). Significant effects after Bonferroni correction with *p* < .025: In panel **(a)**, there is overall a significant stimulus effect with *p* < .001. Within groups, this effect was significant at T0 (IG: *p* = .014, CG: *p* = .001) and at T2 in CG (*p* = .002). In panel **(b)**, there was a significant three-way interaction with *p* = .016.

Figure 3 displays the results from the cued exploration paradigm. Concerning the initial fixation position (%), the model yielded a significant stimulus effect (overall *Wald Χ*^*2*^ (1) = 19.25, *p* < 0.001), indicating that both groups fixated the food stimuli initially more often as the neutral stimuli. Taking a closer look at the parameter estimates, this stimulus effect was only significant for both groups at T0 (IG: *Wald Χ*^*2*^ (1) = 6.07, *p* = 0.014, B = − 13.02, CI = − 23.38 to − 2.66; CG: *Wald Χ*^*2*^ (1) = 12.10, *p* = 0.001, B = − 16.89, CI = − 26.41 to − 7.38). At T1 and T2, the stimulus differences were reduced and not significant in IG (T1: *Wald Χ*^*2*^ (1) = 1.82, *p* = 0.178; T2: *Wald Χ*^*2*^ (1) = 1.62, *p* = 0.203). The stimulus differences in CG remained significant only at T2 after Bonferroni correction (T1: *Wald Χ*^*2*^ (1) = 4.65, *p* = 0.031, B = − 13.89, CI = − 26.52 to − 1.27; T2: *Wald Χ*^*2*^ (1) = 9.63, *p* = 0.002, B = − 17.36, CI = − 28.36 to − 6.40). No other significant main or interaction effects emerged (all *p* > 0.05).

Concerning dwell time (%), overall no significant effects emerged, but a significant three-way interaction (*Wald Χ*^*2*^ (1) = 5.78, *p* = 0.016, B = 8.50, CI = 1.57 to 15.42) indicating that IG increased the dwell time on neutral stimuli from T0 to T1 in contrary to CG, whereas they did not differ in the dwell time on food stimuli. Concerning the neutral stimuli, there was a group x time interaction from T0 to T1 that was not significant after Bonferroni correction (*Wald Χ*^*2*^ (1) = 4.46, *p* = 0.035, B = 6.52, CI = 0.47 to 12.57). Further, CG decreased the dwell time on food stimuli from T0 to T2, which was also not significant after Bonferroni correction (*Wald Χ*^*2*^ (1) = 4.53, *p* = 0.033, B = 5.08, CI = 0.40 to 9.75). No other significant main or interaction effects emerged (all *p* > 0.05).

### Modified antisaccade paradigm

**Figure 4:**
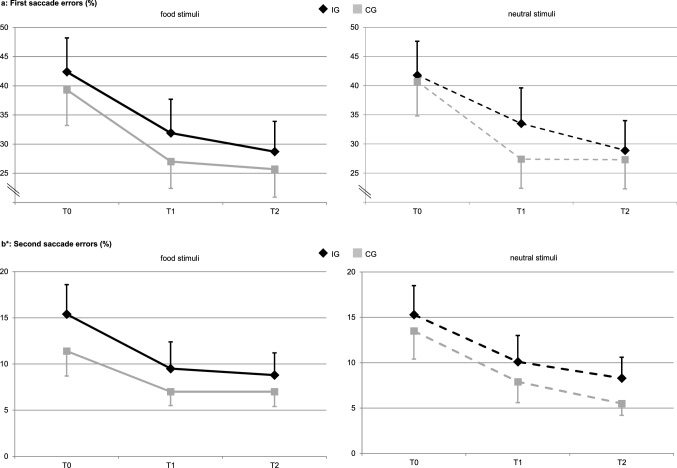
Eye tracking data of the modified antisaccade paradigm. Panel **(a)**: M (SE) of the first saccade errors (%), and Panel **(b)**: M (SE) of the second saccade errors (%) for food and neutral stimuli by treatment group (IG, CG) and measurement point (T0, T1, T2). Significant effects after Bonferroni correction with *p* < .025: in panel **(a)**, there is overall a significant time effect with *p* < .001. In IG, this effect was significant at T2 vs. T0 (food stimuli: *p* = .007, neutral stimuli: *p* = .008). In CG, this effect was significant at T1 vs. T0 (food stimuli: *p* = .003, neutral stimuli: *p* < .001) and at T2 vs. T0 (food stimuli: *p* < .001, neutral stimuli: *p* = .005). In panel **(b)**, there is overall a significant time effect with *p* < .003. In IG, this effect was significant at T1 vs. T0 (food stimuli: *p* = .018, neutral stimuli: *p* = .015). In CG, this effect was significant for neutral stimuli at T1 vs. T0 (*p* = .017) and at T2 vs. T0 (*p* = .004). **(b)** displays the original values though the General Estimating Equations for the second saccade errors (%) were computed with logarithmised values to achieve normal distribution. There is a small inconsistency between **(b)** and the text concerning the second saccade errors (%) in IG: Measurement point T2 has a smaller mean under the food and the neutral stimuli condition as compared to measurement point T1, but only the reduction from T0 to T1 does achieve significance in the GEE model. This is due to the change to the log scale in the model. Concerning the food stimuli, this is additionally due to the change from a descriptive parameter based on unweighted observations to a model parameter derived from weighted observations with weights determined by the working correlation of the GEE model (exchangeable structure).

Figure 4 displays the results from the modified antisaccade paradigm. Concerning the first saccade errors (%), a significant time effect elapsed (overall *Wald Χ*^*2*^ (2) = 18.47, *p* < 0.001), indicating that both groups reduced first saccade errors towards the food and neutral stimuli over both measurement points. More precisely, this time effect was significant at T1 and T2 compared to T0 for both stimuli categories in CG, i.e. for food stimuli at T1 (*Wald Χ*^*2*^ (1) = 9.00, *p* = 0.003, B = − 12.73, CI = − 21.06 to − 4.42) and at T2 (*Wald Χ*^*2*^ (1) = 12.41, *p* < 0.001, B = − 12.56, CI = − 19.55 to − 5.57) as well as for neutral stimuli at T1 (*Wald Χ*^*2*^ (1) = 14.86, *p* < 0.001, B = − 13.79, CI = − 20.8 to − 6.78) and T2 (*Wald Χ*^*2*^ (1) = 7.98, *p* = 0.005, B = − 12.41, CI = − 21.01 to − 3.80). For IG, this time effect was only significant at T2 (food stimuli: *Wald Χ*^*2*^ (1) = 7.15, *p* = 0.007, B = − 9.17, CI = − 15.89 to − 2.45; neutral stimuli: *Wald Χ*^*2*^ (1) = 7.06, *p* = 0.008, B = − 8.26, CI = − 14.35 to − 2.17), but not at T1 (food stimuli: *Wald Χ*^*2*^ (1) = 2.97, *p* = 0.085; neutral stimuli: *Wald Χ*^*2*^ (1) = 0.75, *p* = 0.388). There was a group x time interaction from T1 to T0 concerning neutral stimuli, indicating that CG reduced first saccade errors on neutral stimuli, but not IG. This interaction did not reach significance after Bonferroni correction (*Wald Χ*^*2*^ (1) = 4.10, *p* = 0.043, B = − 10.53, CI = − 20.72 to − 0.34). No other significant main or interaction effects occurred (all *p* > 0.05).

Concerning second saccade errors (%), there was also a significant time effect (overall *Wald Χ*^*2*^ (2) = 11.61, *p* = 0.003), indicating a reduction of second saccade errors in both groups over both measurement points. However, this time effect was only significant for the food stimuli in IG from T0 to T1 (*Wald Χ*^*2*^ (1) = 5.62, *p* = 0.018, B = − 0.226, CI = − 0.413 to − 0.039), whereas the reduction from T0 to T2 in IG failed significance (*Wald Χ*^*2*^ (1) = 3.11, *p* = 0.078). In CG, there was no significant change concerning food stimuli at both time points (T1: *Wald Χ*^*2*^ (1) = 1.47, *p* = 0.225; T2 *Wald Χ*^*2*^ (1) = 1.47, *p* = 0.225). Concerning the neutral stimuli, IG reduced second saccade errors from T0 to T1 (*Wald Χ*^*2*^ (1) = 5.91, *p* = 0.015, B = − 0.236, CI = − 0.426 to − 0.046), but not from T0 to T2 (*Wald Χ*^*2*^ (1) = 2.66, *p* = 0.103). CG reduced those errors towards neutral stimuli from T0 to T1 (*Wald Χ*^*2*^ (1) = 5.68, *p* = 0.017, B = − 0.268, CI = − 0.488 to − 0.047) and from T0 to T2 (*Wald Χ*^*2*^ (1) = 8.34, *p* = 0.004, B = − 0.339, CI = − 0.568 to − 0.109). No other significant main or interaction effects occurred (all *p* > 0.05).

### Stimulus rating

Table 2 shows the results from the stimulus rating. Concerning the pleasantness of food stimuli, a group x time interaction (overall *Wald Χ*^*2*^ (1) = 6.64, *p* = 0.036) was not significant after Bonferroni correction, and a significant time effect elapsed (overall *Wald Χ*^*2*^ (2) = 15.04, *p* = 0.001). More precisely, the interaction was significant for T1 compared to T0 (*Wald Χ*^*2*^ (1) = 6.51, *p* = 0.011, B = 0.68, CI = 0.16 to 1.19), but not for T2 compared to T0 (*Wald Χ*^*2*^ (1) = 2.36, *p* = 0.125). The time effect was only significant in IG at both measurement points (T1: *Wald Χ*^*2*^ (1) = 14.63, *p* < 0.001, B = − 0.78, CI = − 1.18 to − 0.38; T2: *Wald Χ*^*2*^ (1) = 16.17, *p* < 0.001, B = − 0.83, CI = − 1.23 to − 0.42), but not significant in CG (T1: *Wald Χ*^*2*^ (1) = 0.36, *p* = 0.548; T2: *Wald Χ*^*2*^ (1) = 2.19, *p* = 0.139). These results indicate that IG perceived food as less pleasant after the treatment as compared to CG.

**Table 2:** Stimuli ratings (M, SE) of the food and neutral stimuli by treatment group (IG, CG) and measurement point (T0, T1, T2).

	IG			CG		
	T0 (N = 29)	T1 (N = 29)	T2 (N = 28)	T0 (N = 24)	T1 (N = 24)	T2 (N = 24)
**Food stimuli**						
Pleasantness	1.7 (0.3)	0.8(0.3)	0.7 (0.3)	1.2 (0.3)	1.1 (0.3)	0.9 (0.2)
Palatability	1.7 (0.3)	0.7 (0.3)	0.7 (0.3)	1.3 (0.3)	1.1 (0.3)	0.8 (0.3)
Wanting	− 0.2 (0.4)	− 1.7 (0.4)	− 2.0 (0.4)	− 1.8 (0.5)	− 2.4 (0.4)	− 2.3 (0.4)
Liking	2.0 (0.2)	1.4 (0.2)	1.4 (0.2)	1.8 (0.2)	1.5 (0.2)	1.6 (0.2)
Neutral stimuli*						
Pleasantness	0.0 (0.2)	0.2 (0.1)	0.1 (0.1)	0.2 (0.3)	0.6 (0.4)	0.4 (0.3)

The food and neutral stimuli were rated on a Likert scale ranging from − 5 to + 5.

Significant effects are presented in the main text.

*The table displays the original values though the General Estimating Equations for the neutral stimuli were computed with symmetrically logarithmised values to achieve normal distribution.

Concerning the palatability, similar results elapsed with a significant group x time interaction (overall *Wald Χ*^*2*^ (1) = 7.64, *p* = 0.022) and a significant time effect (overall *Wald Χ*^*2*^ (2) = 23.82, *p* < 0.001). The interaction was significant for T1 compared to T0 (*Wald Χ*^*2*^ (1) = 7.23, *p* = 0.007, B = 0.65, CI = 0.18 to 1.11), but not for T2 (*Wald Χ*^*2*^ (1) = 1.19, *p* = 0.275). The time effect was significant in IG at both measurement points (T1: *Wald Χ*^*2*^ (1) = 20.63, *p* < 0.001, B = − 0.79, CI = − 1.13 to − 0.45; T2: *Wald Χ*^*2*^ (1) = 22.96, *p* < 0.001, B = − 0.83, CI = − 1.17 to − 0.49), but not in CG at T1 (*Wald Χ*^*2*^ (1) = 0.79, *p* = 0.375) and not at T2 compared to T0 after Bonferroni correction (*Wald Χ*^*2*^ (1) = 5.04, *p* = . 025, B = −0.52, CI = − 0.97 to − 0.07). These results indicate that IG perceived food as less palatable after the treatment as compared to CG.

Concerning the wanting of food stimuli, there was a significant time effect (overall *Wald Χ*^*2*^ (2) = 31.99, *p* < 0.001) that was significant for both groups and both measurement points, i.e. in IG at T1 (*Wald Χ*^*2*^ (1) = 16.57, *p* < 0.001, B = − 1.30, CI = − 1.93 to − 0.67) and T2 (*Wald Χ*^*2*^ (1) = 17.90, *p* < 0.001, B = − 1.53, CI = − 2.24 to − 0.82), and in CG at T1 (*Wald Χ*^*2*^ (1) = 8.96, *p* = 0.003, B = − 0.71, CI = − 1.18 to − 0.25) and at T2 (*Wald Χ*^*2*^ (1) = 7.04, *p* = 0.008, B = − 0.77, CI = − 1.34 to − 0.20). This indicates that both groups experienced reduced wanting over time. Additionally, a group difference at T0 elapsed (*Wald Χ*^*2*^ (1) = 4.90, *p* = 0.027, B = − 1.32, CI = − 2.49 to − 0.15), indicating that CG displayed comparably less desire to eat the presented food stimuli as compared to IG already at the beginning of the study. However, this group difference was not significant after Bonferroni correction.

Concerning patients’ liking of food stimuli, there was also a significant time effect indicating a reduction of liking (overall *Wald Χ*^*2*^ (2) = 10.49, *p* = 0.005), but this effect only reached significance in IG (T1: *Wald Χ*^*2*^ (1) = 7.89, *p* = 0.005, B = − 0.47, CI = − 0.79 to − 0.14; T2: *Wald Χ*^*2*^ (1) = 9.86, *p* = 0.002, B = − 0.48, CI = − 0.78 to − 0.18), and not in CG (T1: *Wald Χ*^*2*^ (1) = 2.85, *p* = 0.092; T2: *Wald Χ*^*2*^ (1) = 1.61, *p* = 0.205).

Concerning the neutral stimuli, no significant main effects or interactions emerged, besides one time effect in CG from T1 in comparison to T0 (*Wald Χ*^*2*^ (1) = 9.72, *p* = 0.002, B = 0.078, CI = 0.029 to 0.127), which is indicating of a small increase of pleasantness in CG after the treatment period. Overall, the neutral stimuli were perceived by both groups as neutral.

### Correlational analyses

Correlational data is presented as a supplement in Table S1. After Bonferroni correction, the pooled initial fixation position and second saccade errors on food stimuli over both groups and all measurement points did not correlate significantly with eating disorder pathology, BMI, trait impulsivity and stimulus ratings (all *p* > 0.00125). The dwell time on food stimuli correlated significantly with all stimulus ratings concerning food stimuli (pleasantness *r* = 0.33, palatability *r* = 0.36, wanting *r* = 0.45, liking *r* = 0.37), and FCQ-S total score (*r* = 0.35), each *p* < 0.00125, but not with EDE-Q, BMI and trait impulsivity scores. The first saccade errors on food stimuli correlated significantly with FCQ-S total score (*r* = 0.29, *p* < 0.00125) and BMI (*r* = 0.31, *p* < 0.00125), but not with EDE-Q, stimulus ratings and trait impulsivity scores.

### Sensitivity analyses

As there was a nearly significant group difference of FCQ-S total score at baseline and as FCQ-S total score correlates with dwell time and first saccade errors, we computed sensitivity analyses where we included FCQ-S total score as a covariate into the GEEs. Concerning dwell time, FCQ-S total score was not significant (*p* = 0.529), but the group x time x stimulus interaction stayed significant with *p* = 0.016. Concerning first saccade errors, FCQ-S total score was also not a significant covariate (*p* = 0.104) and the reported time effect stayed significant (*p* = 0.005).

## Discussion

We present longitudinal data on food-related impulsivity from patients with BED treated in the IMPULS study—the first randomized controlled trial which delivered a group intervention with a special focus on impulsive eating behavior. In terms of our hypotheses, we found mixed results: The attentional bias towards food stimuli was measured in the cued exploration paradigm. Concerning initial fixation position, this bias vanished in IG at T1 and T2 which is represented by the disappearance of significant stimulus effects. However, IG did not differ from CG and the stimulus effect in CG was not significant after Bonferroni correction at T1 as well. Further, there was a significant group x time x stimulus interaction in dwell time that was mainly driven by an increase of dwell time on neutral stimuli at T1 in IG as compared to CG. However, there was no reduced attentional bias in IG at T2. The inhibitory control deficits, measured by the modified antisaccade paradigm, were contrary to our primary hypothesis: Saccadic errors were overall reduced in both groups and for both stimulus categories, which might indicate a training effect. The first saccade errors on food and neutral stimuli were even more rapidly reduced in CG at T1 when compared to IG, which showed significant reductions only at T2. The second saccade errors, however, were reduced towards food stimuli only in IG at T1, while both groups reduced second saccade errors towards neutral stimuli. The results from the stimuli ratings include reduced pleasantness, palatability, wanting and liking of food at T1 and T2 in both groups. This effect was most pronounced in IG after treatment.

These results suggest a mixture of training and treatment effects which we interpret in the following way: Initially, both groups showed a food-related attentional bias in the cued exploration paradigm at T0 as they fixated more often on food stimuli in comparison to neutral stimuli. In IG, this bias vanished after the IMPULS treatment and at the three months follow-up. This could be due to an ongoing treatment effect, where IG patients perceive food stimuli as less attractive. In contrast, there was only a moderate decrease of this bias observed in CG patients after the treatment period and not at the follow-up. This could display a short-term effect in CG due to increased motivation of change while participating in the trial. Moreover, in contrast to CG, IG showed increased dwell time on neutral stimuli vs. food stimuli at T1. This reflects the patients’ attempt to pay more attention to neutral stimuli instead of food. This finding could represent a treatment effect resulting from a decrease in reward sensitivity after food cue exposure, which was part of the IMPULS treatment.

In the antisaccade paradigm, both groups reduced first and second saccade errors on food and neutral stimuli, indicating increased inhibitory control. CG patients achieved improvements concerning first saccades somewhat faster than IG at T1, whereas IG improved only at T2. Concerning second saccades towards food stimuli, only IG improved at T1, whereas both groups improved concerning neutral stimuli. Thus, the results from the antisaccade task reflect training effects on inhibitory control from the laboratory task itself on the one hand—on the other hand, they might also indicate that IG patients were actively trying to avoid eye movements towards food stimuli. This is in line with the interpretation of the data from the cued exploration paradigm.

Beyond training effects, the reduced impulsive gaze behavior in CG seemed consistent with the observed short-term amelioration of eating disorder pathology in the IMPULS trial^24^. We interpreted this clinical amelioration in our previous paper ^24^ in terms of a short-term effect evoked by the weekly self-observations the patients made. Our current data concerning reduced saccade errors and reduced initial fixations on food stimuli directly after treatment in CG match this interpretation. Moreover, the reduced saccade errors in CG are not food-specific. This is in line with the self-observation protocol, as patients were instructed to monitor other impulsive behavior in addition to binge eating episodes as well. Thus, the eye tracking data could display a more general learning effect on impulsive behavior in CG as a result from these self-observations.

In addition to the eye tracking data, the stimulus ratings indicate that both groups reported reduced wanting and liking of food stimuli over time. However, specifically IG patients rated the food stimuli as less pleasant and palatable as compared to CG. This corresponds to the eye tracking data and could represent a normalized reward sensitivity concerning food stimuli after treatment which is the goal of the IMPULS treatment. Normalized eating behavior indicates a well-balanced and regular diet, which is neither too impulsive nor too restrictive.

The assumption that we measured food-related reward sensitivity with our eye tracking paradigms is further supported by the medium–high correlations of dwell time with the stimulus ratings on food stimuli as well as the food craving total score. First saccade errors on food stimuli correlated with the food craving total score as well. According to these observed correlations, current food craving as a cognitive component of impulsivity might be one part of the underlying mechanisms that are measured by the two eye tracking paradigms. This is in line with findings from the systematic review from Maxwell et al.^43^ It is also worth noting that eye tracking variables did not correlate with EDE-Q, BIS-15 and BIS/BAS, which implies an association to food-related reward sensitivity and not to trait impulsivity or general eating pathology. This is in line with Sharma et al.’s^19^ meta-analytic results, where only small correlations were found between self-reported trait impulsivity and laboratory impulsivity tasks.

Last, first saccade errors correlated additionally with BMI. Concerning BMI, no time effects occurred in our main paper^24^, though our sample was highly obese. Meanwhile, there is several evidence that reward sensitivity or food craving is also increased in obese patients^9^. Recent reviews report that several neuroendocrine signals regulate homeostatic, hedonic and inhibitory brain activity to food cues and that this is altered in patients with obesity^44,45^. Thus, in a more transdiagnostic view, treatments that are focusing on food-related impulsivity might also be helpful for patients who have similar underlying mechanisms such as patients with obesity or bulimia nervosa.

In terms of the limitations, a general point of criticism is that the paradigms implemented here might not have sufficiently displayed the two factors of impulsivity. For instance, in basic research there is currently a debate concerning the validity of the “impulsivity” construct going on^20^. Nevertheless, previous evidence supports both paradigms as reliable and valid e.g.^4,26,46^. Moreover, the correlations mentioned above support that we assessed food-related reward sensitivity. Other self-report instruments of impulsivity, e.g. UPPS^47^ that takes the emotional aspects of impulsive behavior into account, could be associated with the eye tracking outcomes as well. The UPPS includes a subscale called negative urgency, which implies that impulsive behaviors are more likely to be executed in highly emotional states. This is in line with Gullo and colleagues^12^, who proposed emotional impact as a third factor of impulsivity. Additionally, there is meanwhile some evidence that binge eating is triggered by negative emotional states^48^. In terms of the stimulus ratings, it cannot be ruled out that desirability bias might have influenced the results. Furthermore, we did not analyze subgroups of successful vs. unsuccessful treatment completers such as Balodis et al.^49^. Finally, this current study is a subproject of the IMPULS trial^24^. Thus, we were only able to determine which effect size would have been required to detect significant effects with the given sample size, i.e. 69 patients out of the 80 patients initially recruited for the IMPULS trial.

Taken together, the diminished gaze onto food stimuli in IG seems to particularly be due to reduced food-related reward sensitivity, representing a treatment effect. Treatments which reduce food-related impulsivity, i.e. increase self-control, self-efficacy, building intentions and planning might lead to healthier eating behavior^50^. The reduction of the saccade errors in both groups might represent a training effect and indicate that response inhibition skills are more easily malleable than reward sensitivity. The observed training effects might be used in neurocognitive training programs to reduce eating disorder pathology as recommended by Turton and colleagues^51^ , which have already been initiated^52^ and piloted^53^. In conclusion, results from this study suggest that food-related impulsivity measured by laboratory tasks could be reduced by clinical treatments such as the IMPULS program^24^. Such treatments seem to be effective in modifying the underlying impulsive mechanisms of BED.

## Supplementary Information

Supplementary information.

## Data Availability

The datasets analyses during the current study are available from the corresponding author on reasonable request.
