# Supplementary material for: Food-related impulsivity assessed by longitudinal laboratory tasks is reduced in patients with binge eating disorder in a randomized controlled trial
Source: Sci Rep. 2021 Apr 15;11:8225. doi: 10.1038/s41598-021-87231-w (PMC8050257; doi:10.1038/s41598-021-87231-w)
Supplement: Supplementary file 1 — Supplementary information. [file 41598_2021_87231_MOESM1_ESM.docx]

**Food-related impulsivity assessed by longitudinal laboratory tasks is reduced in patients with binge eating disorder in a randomized controlled trial**

Kathrin Schag (kathrin.schag@med.uni-tuebingen.de)^1,2*^

Elisabeth J. Leehr (leehr@uni-muenster.de)^3^

Paolo Meneguzzo (meneguzzo.p@gmail.com)^4^

Peter Martus (peter.martus@med.uni-tuebingen.de)^5^

Stephan Zipfel (stephan.zipfel@med.uni-tuebingen.de)^1,2^

Katrin E. Giel (katrin.giel@med.uni-tuebingen.de)^1,2^

^1^ Medical University Hospital Tübingen, Department of Psychosomatic Medicine and Psychotherapy, Tübingen, Germany

^2^ Competence Center for Eating Disorders Tübingen (KOMET), Tübingen, Germany

^3^ University of Münster, Department of Psychiatry and Psychotherapy, Münster, Germany

^4^ University of Padova, Department of Neurosciences, Padova, Italy

^5^ Medical University Hospital Tübingen, Institute for Clinical Epidemiology and Applied Biostatistics, Tübingen, Germany

*** Corresponding author:**

Kathrin Schag, Medical University Hospital Tübingen, Department of Psychosomatic Medicine and Psychotherapy, Osianderstraße 5, 72076 Tübingen, Germany

E-Mail: kathrin.schag@med.uni-tuebingen.de

Phone: +491-7071-29-89117

**Table S1.** Correlations between the eye tracking variables concerning food stimuli and indices of eating behaviour and impulsivity pooled for study arm and measurement points. N ranging from 146 to 151 depending on the correlated variables.

|  | **EDE-Q total score** | **FCQ-S total score** | **BMI (kg/m²)** | **Pleasantness** | **Palatability** | **Wanting** | **Liking** | **BIS-15 total score** | **BIS total score** | **BAS total score** |
| --- | --- | --- | --- | --- | --- | --- | --- | --- | --- | --- |
| **initial fixation position on food stimuli (%)** | .07 | .13 | -.20* | .22* | .13 | .20* | .13 | .01 | .00 | -.08 |
| **Dwell time on food stimuli (%)** | .11 | **.35***** | -.11 | **.33***** | **.36***** | **.45***** | **.37***** | .02 | -.01 | .03 |
| **first saccade errors on food stimuli (%)** | .24* | **.29***** | **.31***** | .11 | .13 | .22* | .03 | -.08 | .08 | -.03 |
| **second saccade errors on food stimuli (%)** | .12 | .15 | .06 | .02 | .05 | .10 | .04 | -.06 | .09 | .01 |

**Notes.** BAS, Behavioral Activation System; BIS, Behavioral Inhibition System; BIS-15, Barratt Impulsiveness Scale (short version); EDE-Q, Eating Disorder Examination Questionnaire; FCQ-S, Food Craving Questionnaire State.

The food stimuli were rated on Likert scales ranging from -5 to +5 concerning pleasantness, palatability, wanting and liking.

* p < .05, ***** p < .00125**
